# No Impact of Deep Brain Stimulation on Fear-Potentiated Startle in Obsessive–Compulsive Disorder

**DOI:** 10.3389/fnbeh.2014.00305

**Published:** 2014-09-09

**Authors:** Johanna M. P. Baas, Floris Klumpers, Mariska H. Mantione, Martijn Figee, Nienke C. Vulink, P. Richard Schuurman, Ali Mazaheri, Damiaan Denys

**Affiliations:** ^1^Department of Experimental Psychology, Faculty of Social Sciences, Utrecht University, Utrecht, Netherlands; ^2^Helmholtz Institute, Utrecht, Netherlands; ^3^Department of Cognitive Neuroscience, Donders Institute for Brain, Cognition and Behavior, Radboud University, Nijmegen, Netherlands; ^4^Department of Psychiatry, Academic Medical Center, University of Amsterdam, Amsterdam, Netherlands; ^5^Department of Neurosurgery, Academic Medical Center, University of Amsterdam, Amsterdam, Netherlands; ^6^Netherlands Institute for Neuroscience, Royal Netherlands Academy of Arts and Sciences, Amsterdam, Netherlands

**Keywords:** deep brain stimulation, obsessive–compulsive disorder, fear-potentiated startle, context, bed nucleus of the stria terminalis

## Abstract

Deep brain stimulation (DBS) of the ventral internal capsule is effective in treating therapy refractory obsessive–compulsive disorder (OCD). Given the close proximity of the stimulation site to the stria terminalis (BNST), we hypothesized that the striking decrease in anxiety symptoms following DBS could be the result of the modulation of contextual anxiety. However, the effect of DBS in this region on contextual anxiety is as of yet unknown. Thus, the current study investigated the effect of DBS on contextual anxiety in an experimental threat of shock paradigm. Eight patients with DBS treatment for severe OCD were tested in a double-blind crossover design with randomly assigned 2-week periods of active and sham stimulation. DBS resulted in significant decrease of obsessive–compulsive symptoms, anxiety, and depression. However, even though the threat manipulation resulted in a clear context-potentiated startle effect, none of the parameters derived from the startle recordings was modulated by the DBS. This suggests that DBS in the ventral internal capsule is effective in treating anxiety symptoms of OCD without modulating the startle circuitry. We hypothesize that the anxiety symptoms present in OCD are likely distinct from the pathological brain circuits in defensive states of other anxiety disorders.

## Introduction

Obsessive–Compulsive Disorder (OCD) is an anxiety disorder characterized by persistent thoughts (obsessions) and repetitive ritualistic behaviors (compulsions). Treatments for OCD consist of cognitive-behavioral therapy and pharmacotherapy with serotonin reuptake inhibitors. Even when the best available treatments are applied approximately 10% of all patients remain severely affected (Denys, 2006). For a proportion of these patients, deep brain stimulation (DBS) may be a treatment option (Denys and Mantione, 2009).

Deep brain stimulation is a neurosurgical treatment involving programmable electrical stimulation of brain tissue through electrodes implanted in specific locations of the brain. DBS is effective in patients with treatment-refractory OCD when aimed at the anterior limb of the internal capsule, the ventral striatum, the nucleus accumbens (NAcc), or the subthalamic nucleus (Nuttin et al., 1999, 2003; Sturm et al., 2003; Abelson et al., 2005; Greenberg et al., 2006, 2010; Mallet et al., 2008). In a controlled trial (Denys et al., 2010), it was shown that bilateral stimulation targeted at the NAcc, with the active points of stimulation in the ventral part of the anterior internal capsule just adjacent to the accumbens (van den Munckhof et al., 2013) can be an effective treatment in highly refractory OCD patients.

The current study investigated if the reduction in anxiety experienced by DBS treatment in OCD patients is dependent on changes in the basic defense systems in the brain indexed by fear-potentiated startle (FPS). The stimulation site is near to and has connections with the bed nucleus of the stria terminalis (BNST). The BNST is part of the extended amygdala, and has its largest cell concentration surrounding the anterior commissure bilaterally (de Olmos and Heimer, 1999; Mai et al., 2003). Preclinical work has established a dissociation between phasic fear responses to specific threats (orchestrated by the amygdala) and contextual anxiety, which is a more sustained state in anticipation of potential threats that relies on the BNST (Walker et al., 2003; Sullivan et al., 2004; Davis et al., 2010). Human neuroimaging studies have confirmed activation of this area in states of anticipatory anxiety (Straube et al., 2007), especially in the context of sustained unpredictable threat (Alvarez et al., 2011). The involvement of the BNST in contextual anxiety may imply that the therapeutic efficacy observed in OCD with DBS of anatomical areas near or connected to the BNST involves anxiolysis through modulation of contextual anxiety generated by the BNST. Alternatively, a recent study in rodents suggests that the treatment effect of DBS at this location could involve extrastriatal effects, involving areas known to be involved in the modulation (especially extinction) of fear responses (Rodriguez-Romaguera et al., 2012). The double-blind cross over DBS protocol allowed a unique opportunity to assess acute effects of DBS on reactivity of human defense states.

To this end, we employed an experimental threat of shock paradigm in combination with FPS recordings. The design was developed to dissociate states of fear and anxiety that were shown to be dependent on different neural substrates in animal models (Davis et al., 2010). Cued fear, presumably dependent on the amygdala, was elicited by means of a predictable shock condition and contextual anxiety, presumably dependent on the BNST (Alvarez et al., 2011) was evoked by the general experimental context and an unpredictable shock condition (Grillon et al., 2004). Patients who were stabilized several months after having been implanted with a deep brain stimulator were tested in a randomized on/off protocol. Our rational was that if DBS did indeed influence contextual anxiety, turning off the stimulation would result in elevated baseline startle in addition to increased context-potentiated startle. In contrast, we expected cued FPS to be unaffected. Because some studies have assessed startle reactivity in patients with OCD, but none of those involved startle reactivity in the context of a threat [see Grillon and Baas (2003) for a review of the importance of this factor], a comparison with a group of healthy control subjects who underwent the same threat of shock, startle modulation protocol was also included.

## Materials and Methods

### Ethics statement

All patients consented to participate in this study and the experimental procedures were approved by Medical Ethics Committee of the Academic Medical Center, University of Amsterdam. The trial was registered under trial number ISRCTN23255677 in the international controlled trial registry.

### Patients

Eight patients from a clinical trial reported in reference Denys et al. (2010) participated in the startle protocol. All patients were diagnosed as having primary OCD according to DSM-IV criteria using the Structured Clinical Interview for DSM-IV Axis I disorders (First et al., 1997). Due to logistical and technical difficulties, not all patients completed the FPS test in all three phases of the protocol. The patient demographics, double-blind counterbalancing of the DBS on/off along with missing sessions are summarized in Table 1. Patient characteristics of this sample were: 4 male, 4 female, mean age 39.2 (SD 11.1), mean Y-BOCS scores prior to start of DBS treatment 34.0 (SD 3.7) and after having been stabilized on DBS treatment 13.1 (SD 10.6), mean percentage improvement was 61% (range 7–100%).

**Table 1:** **Patient and session information**.

Patient ID	Sex/age	Axis I comorbidity	Session prior to on/off phase	Double-blind session 1	Double-blind session 2	Notes
2	M/44	MDD	ON	OFF	ON	Baseline startle only
4	F/27	Dysth.	ON	ON	OFF	
5	M/41	MDD	ON	OFF	ON	
8	F/36	–	ON	OFF	x	Last session lost (equipment failure)
10	F/35	–	x	ON	OFF	Pre-session skipped (logistical difficulties)
11	F/46	PD	ON	OFF	ON	
12	M/60	–	ON	ON	OFF	
15	M/57	MDD	x	OFF	ON	Pre-session skipped (logistical difficulties)

*The patient IDs are based on the coding used in Denys et al. (2010). Patient 2 only participated in a baseline startle measurement. Sessions for which no data are available are marked with an “x”*.

*MDD, major depressive disorder; Dysth., dysthymia; PD, panic disorder*.

### Treatments

The study was part of a larger protocol in which the patients underwent surgery for implementation of the deep brain stimulator and follow up assessments (Denys et al., 2010). The DBS electrodes were four direct-contact electrodes (Medtronic, Inc., Minneapolis, Minnesota; contact points 1.5-mm long, separation 0.5 mm) implanted bilaterally. Target coordinates for the deepest electrode contact were 7 mm lateral to the midline, 3 mm anterior to the anterior border of the anterior commissure, and 4 mm inferior to the intercommissural line. This study took place after patients had been stabilized on the DBS treatment for 8 months [refer to Denys et al. (2010), for additional information]. Patients were randomly allocated to two periods of 2 weeks with the stimulators blindly turned on (active stimulation) in one period and turned off in the other. The laboratory personnel conducting the startle protocol was blind to stimulation conditions. FPS measurements were planned at three time points surrounding the on–off phase of the protocol: (i) at baseline (i.e., after the 8-month stabilization, stimulator on), (ii) at the end of a 2-week period of active or sham stimulation (right before switching to the other condition), and (iii) at the end of the second 2-week period of reversed active or sham stimulation.

### Stimuli and apparatus

Shock reinforcements were delivered through two disk electrodes located on the inside of the subjects’ forearms. Stimulation level was individually set with a standardized shock work-up procedure. Shocks consisted of a short 2-ms pulse that was delivered with a Digitimer DS7A constant current stimulator^1^. The work-up consisted up to five sample shocks, after each of which subjects rated how annoying/painful they found the preceding shock on a five-point scale. The level used in the study corresponded with a rating of four out of five (“quite a bit painful/annoying”). There was no systematic difference between the DBS on and off sessions in shock intensity (*t* < 1, NS).

The startle reflex was evoked by bursts of white noise presented through headphones with 50-ms duration and an intensity of 105 dB(A). Eye blink electromyography (EMG) was measured with two electrodes on the lower orbicularis oculi with a Biosemi system^2^. Task conditions and visual analog scales (VAS) were presented on a computer screen by automated scripts (Presentation)^3^. Patients were tested using laboratory equipment set up in a dedicated room at the University Medical Center of Utrecht University or the Academic Medical Center of the University of Amsterdam, the Netherlands.

### Experimental procedure

Clinical rating scales for Obsessive–Compulsive symptoms (Y-BOCS), depressive symptoms (HAM-D), and anxiety (HAMA) were assessed prior to the FPS procedure The FPS procedure started with placement of electrodes on the *orbicularis oculi* muscle for startle measurement. The design of the current study allowed assessment of several levels of contextual anxiety. First, baseline startle measurements were taken (two series of nine startle probes presented with average intervals of 16 s). Then, the shock work-up procedure to determine the shock levels for the FPS experiment was performed. Instructions concerning the FPS experiment were given, after which another series of nine startle probes was presented. The FPS experiment followed immediately after the last habituation phase, and consisted of three contexts in which instructions regarding possible shock administration varied (cf. Grillon et al., 2004). The three contexts were signaled by written instructions displayed on a computer monitor. Instructions were: “No shock” (Neutral context, N); “Shock only during cue” (Predictable context, P); and “Shock at any time” (Unpredictable context, U). In each context, cues were presented, e.g., red square for N, blue circle for P, and green triangle for U (counterbalanced between subjects). These cues were only predictive of a possible shock in the P context. Duration of contexts was 90–100 s, during which the written instructions concerning shock delivery remained on the screen and four cues were presented at regular time intervals with 8-s duration. Startle responding was assessed during all experimental conditions, i.e., during the presentation of these cues and in their absence to measure responses to the context. The FPS test consisted of two blocks with the following predetermined orders of contexts: (1) P–N–U–N–U–N–P and (2) U–N–P–N–P–N–U. Each session consisted of both blocks, with the order of these two blocks counterbalanced across subjects. During each context, three startle probes were delivered in the absence and three in the presence of a cue, hence 6 per experimental condition per block and 12 in total across the experiment. Intervals between startle probes varied between 12 and 18 s (16 s on average). In each block, one of the two occurrences of the shock (P and U) contexts contained one shock reinforcement, and the other occurrence contained two shock reinforcements (12 shocks per test session). Shock reinforcements during P contexts always coincided with a cue, hence predictably, while reinforcements during U were administered in absence of the cue. Orders of startle trials and shock reinforcements were varied between blocks, sessions, and subjects. Each block began with three startle probes for habituation.

After each block, subjective ratings on levels of fear specified for all experimental conditions were collected using computerized VAS scales (anchors 0 = not fearful, 100 = very fearful), as well as ratings on subjective state at that time, including fearfulness (same anchors), calmness (anchors 0 = not at all calm, 100 = very calm), alertness (anchors 0 = not alert, 100 = very alert), and need for control (anchors 0 = no need, 100 = strong need).

### Data analyses

Analysis of the EMG signal was carried out in Brain Vision Analyzer software^4^ according to established procedures (Blumenthal et al., 2005). Interference of electrical activity coming from the DBS stimulator affected the EMG measurements. This interference was removed by filtering the data with band rejection filters aimed at the particular frequency of that individual patient’s DBS stimulator (most often 186 Hz and harmonics up to 744 Hz). The session in which the stimulator was off was processed with the same filter settings per patient. Filtered data blinded with respect to DBS condition were visually inspected and trials containing excessive noise in the baseline were removed. Patient 15 had relatively noisy data, hence analyses excluding this patient were also performed. Then, data were further processed using automated scripts in which they were band pass filtered according to startle guidelines (28–300 Hz), rectified, and smoothed with a low pass filter at 24 Hz (24 dB/oct). Startle magnitude was defined as the peak of the smoothed signal in the period between 25 and 125 ms after onset of the acoustic startle probe.

All reported analyses were based on normalized data to reduce between-subjects variance in baseline startle. There was no difference in statistical results with the raw data analyses. *Z*-scores were calculated across all measurements per patient and converted to *T*-scores [(Z*10) + 50]. Averages per session and condition across trials were calculated. To use all available data, data from the two “on” sessions were averaged together for patients who participated in a baseline assessment in addition to an “on” session in the crossover phase Pattern of results from the baseline and double-blind “on” sessions did not differ and results were the same for analyses in which only the blinded conditions were included. Order of on/off sessions was balanced across patients with four patients starting with “on” and four patients starting with “off”.

### Statistical analysis

All statistical analyses were univariate repeated measures analyses of variance performed in SPSS 16.0 for Windows. Statistical tests for the habituation phase focused on the startle habituation series before and after the shock workup separately with factors DBS (on, off) × Block. The factor Block consisted of averages of three subsequent startle trials, and contained six levels in the series before and three levels in the series after the shock workup. For threat modulation of startle, a DBS (on, off) × Context (neutral, predictable, unpredictable) × Cue (intertrial interval, cue) repeated measures analyses was conducted. In addition, specific tests were aimed at DBS on/off effects on FPS and subjective fear evoked by cued threat and unpredictable shock. These consisted of Cue (absent, present) × DBS (on, off) and Context (neutral, unpredictable) × DBS (on, off) repeated measures ANOVAs. An additional ANOVA tested for differences with normalized startle modulation data from a control group [placebo session taken from reference Baas et al. (2009)] with repeated measures for the task conditions and group as a between subject factor for the on and off sessions separately.

## Results

### Effects of treatment on mood and anxiety

Switching the stimulator off during the cross over phase resulted in significant increases in OCD symptoms compared to the average across the baseline and “on” session [Y-BOCS on 17.3 (SD 10.8), off 28.5 (SD 10.9), difference *t*(7) = 2.9, *p* = 0.025], anxiety [HAMA on 12.3 (SD 4.7), off 24.3 (SD 8.5), difference *t*(7) = 5.4, *p* = 0.001], and depressed mood [HAM-D on 11.6 (SD 5.5), off 23.8 (SD 9.6), difference *t*(7) = 5.2, *p* = 0.001].

### Baseline startle

**Figure 1:**
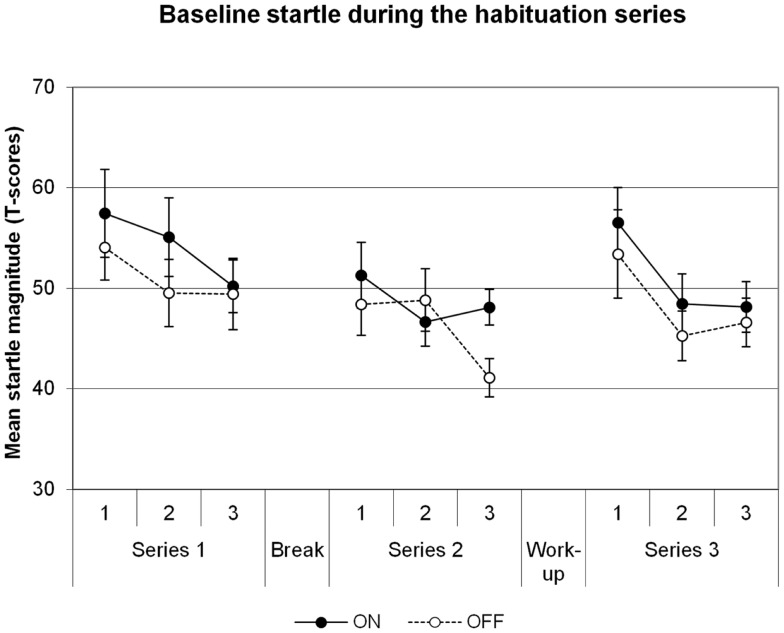
**Baseline startle, data averaged across groups of three subsequent trials in each of the three habituation series**. Displayed are the mean magnitudes with standard errors of the mean.

Baseline startle data are illustrated in Figure 1. There was a significant main effect of Block in the series before the shock workup, indicating habituation of the startle reflex across time [Block *F*(5,35) = 8.2, *p* = 0.001]. However, there was no significant effect of DBS on the startle reflex [main effect for DBS *F*(1,7) = 1.1, *p* = 0.331; DBS × Block *F*(5,35) = 1.5, *p* = 0.231]. The series after the shock workup yielded comparable results: main effect of Block [*F*(2,14) = 20.8, *p* = 0.00006], no effect of DBS [DBS *F*(1,7) = 0.4, *p* = 0.550; DBS × Block *F*(2,14) = 0.2, *p* = 0.815]. To investigate the increases in startle after the shock workup, a specific comparison between the last block before and first block after the workup was made. The workup induced a significant increase in startle in the transition from series 2 to series 3 [*F*(1,7) = 71.5, *p* = 0.00006]. However, this effect did not differ between the DBS conditions [*F*(1,7) = 0.6, *p* = 0.451]. Thus, we find no influence of DBS on baseline startle or due to shock sensitization following the workup.

### Fear-potentiated startle – contexts and cues

**Figure 2:**
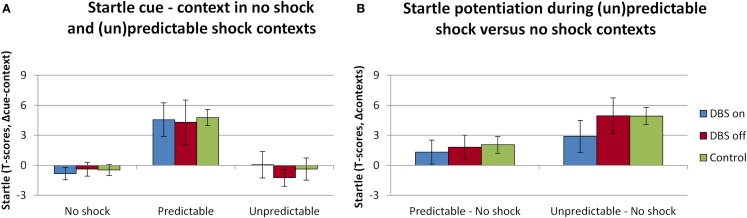
**Significant fear-potentiated startle effects as a result of the threat manipulation were apparent during the predictable context [(A), difference scores cue minus context], and in the comparison between the Neutral and Unpredictable contexts [(B), difference scores (un)predictable minus neutral contexts]**. Displayed are the mean magnitudes with standard errors of the mean. Data from the patient sample are plotted separately for the sessions in which the stimulator was on (plotted in blue) versus off (plotted in red), alongside the startle data from the control sample that was acquired as part of a different study (plotted in green).

The overall analysis of the startle data revealed the expected effects of threat, see Figure 2. The main effects of Context [*F*(2,12) = 9.1; *p* = 0.004] and Cue [*F*(1,6) = 5.7; *p* = 0.064] were (trend level) significant, as well as their interaction [F(2,12) = 7.7; *p* = 0.007]. However, the DBS (on, off) did not interact with any of these factors [DBS × Context *F*(2,12) = 0.4, *p* = 0.681; DBS × Cue *F*(2,12) = 0.1, *p* = 0.722; DBS × Context × Cue *F*(2,12) = 0.2, *p* = 0.822]. Also, as observed in the baseline analysis, the manipulation of switching the DBS on or off did not affect the overall level of startle [*F*(1,6) = 0.3, *p* = 0.596]. More specific contrasts to compare the cue effect in the predictable context (Figure 2A), and the context effect across neutral and unpredictable contexts in absence of the cues (Figure 2B) were performed. Effects of DBS on explicitly cued fear were tested within the predictable condition with factors DBS (on, off) and Cue (absent, present). This test yielded a significant effect of Cue [*F*(1,6) = 9.6; *p* = 0.021] yet no interaction DBS × Cue [*F*(1,6) = 0.007, *p* = 0.937], in line with our hypothesis. The specific test of differences in contextual anxiety effects on startle potentiation between DBS on and off included only the factors Session (DBS on, off) and Context (neutral, unpredictable). This test yielded again a significant effect of Context [*F*(1,6) = 7.7; *p* = 0.033] and a non-significant effect of DBS [DBS × Context *F*(1,6) = 1.4, *p* = 0.281]. It must be noted that the non-significant effects of DBS were very likely not due to lack of power, as the apparent DBS effect on context (U–N) in the figure disappeared almost completely after removing one patient with excessive artifacts, yet the pattern of statistical results remained the same. There were no differences between the patients and the control group taken from a previous study on between-subjects comparisons of cued or contextual startle potentiation (all *t* values <1.4, NS; see green bars in Figure 2).

### Subjective reports – threat manipulation

**Figure 3:**
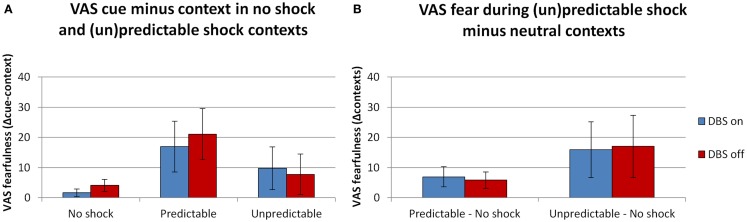
**Ratings of fearfulness during threat manipulation**. Effects of threat were found during the predictable context [**(A)**, difference cue minus context], and in the comparison between the neutral and unpredictable contexts [**(B)**, difference scores (un)predictable minus neutral contexts]. Displayed are the mean magnitudes with standard errors of the mean.

In line with the startle results there were (trend level) significant effects of both the context and cue manipulation. See Figure 3 for the fearfulness ratings during all conditions in the FPS experiment. The overall analysis with the factors DBS (on, off) × Context (neutral, predictable, unpredictable) × Cue (absent, present) × Block (1, 2) revealed (trend level) significant main effects of Context [*F*(2,10) = 4.3, *p* = 0.045] and Cue [*F*(1,5) = 5.2, *p* = 0.071]. Their interaction was not significant [*F*(2,10) = 2.4, *p* = 0.141]. DBS (on, off) did not interact with any of these factors [DBS × Context *F*(2,10) = 0.1, *p* = 0.942; DBS × Cue *F*(2,10) = 2.4, *p* = 0.186; DBS × Context × Cue *F*(2,10) = 1.2, *p* = 0.341]. Specific tests of the cue effect in the predictable condition (Figure 3A) and the context effect (unpredictable – neutral, Figure 3B) between DBS conditions were also not significant [*F*(1,5) < 1.5, NS]. Also, in contrast to the baseline anxiety ratings, DBS on/off did not significantly affect the overall level of fearfulness [*F*(1,5) = 1.6, *p* = 0.257].

### Subjective state

See Table 2 for the assessments of anxiety, need for control, calmness, and alertness. Subjective reports were missing for one patient. Because of the small size of the remaining sample (*n* = 6), effects of trend level significance (*p* < 0.10) are considered meaningful. Ratings of subjective states of anxiety, calmness, alertness, and need for control were collected before the experiment and after each of the habituation blocks (3) and each of the FPS blocks (2).

**Table 2:** **Subjective ratings taken at six moments in between the blocks of the habituation and fear-potentiated startle experiments**.

		Moment											
		Pre		Hab 1		Hab 2		Hab 3		FPS 1		FPS 2	
		Mean	St Err	Mean	St Err	Mean	St Err	Mean	St Err	Mean	St Err	Mean	St Err
Anxious	On	33.3	9.2	45.0	12.3	38.8	12.4	24.9	10.4	52.4	7.4	68.0	11.3
	Off	54.0	9.6	55.2	13.0	55.2	12.0	51.4	16.6	37.2	10.1	52.5	16.0
Need for control	On	37.4	8.5	42.0	13.3	34.5	12.0	38.8	12.7	35.0	12.4	24.8	11.0
	Off	47.0	8.6	49.8	10.8	43.0	10.8	41.0	12.7	49.3	13.6	45.0	15.8
Alert	On	63.7	10.6	53.8	9.9	51.7	8.7	50.9	7.5	35.8	10.4	34.0	11.6
	Off	62.8	12.0	56.8	13.6	42.8	10.5	53.0	16.2	56.5	12.2	34.0	11.4
Calm	On	62.0	4.6	48.4	13.4	56.4	12.0	57.0	13.1	36.6	12.2	26.5	11.8
	Off	40.0	11.6	42.8	13.9	39.0	10.5	67.6	15.4	50.0	10.7	44.0	11.1

*The moments were before start of the session (Pre), after each of the three habituation blocks (Hab 1, 2, 3), and after the two fear-potentiated startle blocks (FPS 1, 2)*.

Only subjective anxiety differed between the DBS on and off conditions, with patients reporting more anxiety throughout the test session when the stimulator was switched off [see Table 2, trend level significant main effect of DBS, *F*(1,5) = 4.9, *p* = 0.078; *F* values for other measures <1.8, NS]. The need for control showed a similar sustained effect, with assessments taken during the stimulator off condition being higher, but this did not result in a (trend level) significant test. Once again there were no interactions between DBS status and the different time points at which the measurements were taken [DBS × Moment for all measures, *F*(1,5) < 1.8, NS].

## Discussion

In our current study, we found clear effects of startle potentiation by threatening cues and contexts, even in the relatively small sample of patients. Moreover, electrical stimulation in the ventral internal capsule, in the proximity of the NAcc had clear effects on subjects’ mood and OCD symptoms. Yet, we found no effects of stimulation on any parameter of baseline startle or context FPS. The threat manipulations also affected subjective fearfulness but again without an effect of DBS. As hypothesized, there were also no effects of electrical stimulation on cued fear. Additionally, an exploratory comparison showed that the startle modulation data from the patients did not differ from the data from a group of healthy individuals assessed in a related study. Taken together, this suggests that the anxiolytic effects observed after DBS treatment in OCD do not involve the neuronal circuits that modulate defensive states in humans.

The lack of effect on the context-potentiation of startle is in contrast to our hypothesis that DBS at this location could affect the BNST through stimulation of the connected ventral anterior internal capsule. Effects of stimulation proved highly dependent upon the exact brain target. As discussed by Denys et al. (2010), response differences of 60–80% were observed depending on which of the four electrode contacts were stimulated, even while these lie as little as 1.5 mm apart. In the treatment protocol in which the patients in this study participated, the site of stimulation that appeared most effective for treatment outcome were the relatively dorsal electrode contacts (Denys et al., 2010). Because the relatively dorsal contacts were anatomically most likely to (partly) overlap with the anatomical locus of the BNST, this provides further support for our initial hypothesis that reduction of contextual fear could potentially have underlain the treatment effects of DBS. However, while the site of stimulation is in close proximity to the BNST, the complete absence of an effect of stimulation here on context-potentiated startle suggests that either the BNST was not sufficiently manipulated with stimulation at this site, or the model in which the BNST is responsible for contextual fear does not accurately describe the role of this area in human defensive responding, despite emerging initial evidence that the BNST is activated during similar states in humans (Alvarez et al., 2011). The validity of this model will have to be further investigated in future studies.

While the observation by Denys et al. (2010) that the more dorsal contacts were more effective is of potential great importance, the significance of the location and the exact effect of stimulation on neuronal tissue remains to be clarified. Relatively, little is known about the exact consequences of stimulating areas of the brain in which so many functional units are tightly packed. In order to delineate the exact mechanisms behind the treatment efficacy more detailed studies of effects of stimulation at different sites within the ventral striatum are needed. These types of procedures could greatly enhance the understanding of the role of different parts of the ventral striatum in anxiety and defense.

Even though the basic startle and FPS effects remained unaffected DBS had a pronounced effect on anxiety symptoms in these patients [see also Denys et al. (2010)]. This suggests that DBS in the ventral internal capsule is effective in treating anxiety symptoms of OCD without modulating the startle circuitry. Effective reduction of symptoms without effects on startle in addition to no difference in startle modulation between patients and controls suggest that the core pathology in OCD may not involve alterations in a basic defensive state that results from the threat of electric shock. In interpreting these findings it may be of importance that OCD is not considered a typical anxiety disorder (Stein et al., 2010). This argument is supported by neurocognitive, neuroimaging, and pharmacotherapy studies that differentiate OCD from other anxiety disorders. OCD patients typically show baseline hyperactivity and increased activity after symptom provocation within the cortico-striatal circuitry, a profile that is absent in neuroimaging studies of patients with other anxiety disorders (Whiteside et al., 2004; van den Heuvel et al., 2005; Etkin and Wager, 2007; Rotge et al., 2008). Distinct neurocognitive deficits related to cortico-striatal circuitry, e.g., impaired motor initiation and execution, have been found in patients with OCD but not in other anxiety disorders (Purcell et al., 1998). Finally, though pharmacological treatment with benzodiazepines is successful in anxiety and related disorders, benzodiazepines do not show efficacy in patients with OCD (Koran et al., 2005). Taken together, evidence of various nature distinguishes OCD from other anxiety disorders.

The unpredictable shock context that we used apparently does not reflect the form of anxiety present in OCD. The unpredictable shock context was designed to evoke a state of longer duration contextual threat (Grillon and Baas, 2003) generated by the BNST as opposed to “fear” responses to short duration cued threats generated by the amygdala [reviewed by Walker et al. (2009)]. Based on this, DBS effects were expected on contextual threat. No effects were expected on cued threat, as abnormalities in amygdala activation have been observed (van den Heuvel et al., 2004; Simon et al., 2010) but not consistently (Cannistraro et al., 2004; Deckersbach et al., 2006), suggesting that they are not at the core of OCD pathology. However, other authors have suggested an alternative distinction between fear and anxiety responses in immediate versus potential threats. Potential threats evoke a set of behaviors that is clearly distinct from immediate threats, including risk assessment (Blanchard et al., 2011) and precautionary behavior (Eilam et al., 2011). A failure to shut down precautionary behaviors has been suggested to underlie the pathology of OCD (Szechtman and Woody, 2004; Boyer and Lienard, 2006). Whereas, threat detection systems including the amygdala and BNST are involved in the initiation of precautionary behaviors (Szechtman and Woody, 2004), the failure to terminate these behaviors may be more related to dysfunctional cortico-striatal loops (Woody and Szechtman, 2011).

The idea that the pathology in OCD involves a failure to terminate risk assessment behavior associated with potential threats rather than responding to threat itself concurs with the present finding that OCD patients did not differ from the healthy control group in startle responding to cued and contextual threat. Interestingly, patients with generalized anxiety disorder (GAD) did also not differ from controls in cued and contextual startle responses (Grillon et al., 2009). Both OCD and GAD have been associated with more psychic or higher cognitive aspects of anxiety such as worrying and rumination, while physiological reactivity to a general stressor is not enhanced (Craske et al., 2009). No previous studies of cued or contextual FPS in OCD have been published to date. Startle studies in the absence of a stressor, e.g., in the context of a pre-pulse inhibition experiment, did not find a general increase in startle reactivity in OCD (Swerdlow et al., 1993; Hoenig et al., 2005; Ahmari et al., 2012). Two studies reported elevated baseline startle in OCD in the context of aversive emotion manipulations (Hoehn-Saric et al., 1995), though in one of these studies this effect disappeared when excluding patients who were on psychotropic medication (Buhlmann et al., 2007). Our study did not find evidence for stress induced hyper reactivity in OCD patients. Taken together, these results support the conclusion that baseline startle and startle increases after general aversive stimulation does not reflect the anxiety present in OCD. However, it cannot be ruled out that OCD patients will show differential modulation of startle when using threats specific to the patients’ anxiety, e.g., pictures of dirty surroundings for patients suffering from fear of contamination.

Our findings are in line with an apparent dissociation between the brain circuitry involved in defensive states in humans (Mobbs et al., 2009; Mechias et al., 2010) and the orbitofrontal–striatal circuit primarily affected in OCD as referred to above (Whiteside et al., 2004). Indeed, only the latter circuit was shown to be affected by DBS treatment of the ventral striatum (Rauch et al., 2006). However, a recently proposed alternative take is that the fear circuitry does play a role in the phenomenology of OCD (Milad and Rauch, 2012). Specifically, these authors propose that the neural circuitry involved in fear expression, such as the amygdala and the dorsal ACC, play a role in OCD, at least in the maintenance of symptoms (Milad and Rauch, 2012). These areas have also been found of importance in instructed fear as evoked in the present study (Klumpers et al., 2010). The idea that stimulation at the ventral striatum may affect this circuitry is supported by a recent study by Rodriguez-Romaguera et al. (2012) of DBS in rodents at ventral striatal sites. They observed enhanced extinction learning when high-frequency stimulation was administered at relatively dorsomedial ventral striatal sites during extinction training. These effects (reduced freezing) were present during extinction training as well as during retrieval the next day. The effects during extinction training could involve a direct effect on fear expression, an interpretation that is not supported by the present data. Alternatively, the observed stimulation-induced plasticity observed at ventral prefrontal sites known to be crucially involved in extinction learning (Rodriguez-Romaguera et al., 2012; Do-Monte et al., 2013) is consistent with no effect in the present study, considering that it does not involve extinction learning. Together, these results very strongly suggest a focus on effects on extinction training in future studies.

The first and foremost limitation of the study concerns the relatively small number of patients (*n* = 8). Yet, this did not prohibit replication of the basic effects of cued and contextual startle potentiation. The relatively small sample also precluded detailed analysis of potential confounds with depressive symptoms, since depression may have opposite effects on FPS than anxiety (Melzig et al., 2007). Moreover, our study employed electric shocks as threatening stimuli. Future studies could use symptom relevant threats, which may prove to elicit differential reactivity in OCD.

In conclusion, DBS in the ventral internal capsule, connected to the BNST, clearly improves anxiety in OCD patients (Denys et al., 2010), but does not affect contextual anxiety or other startle parameters in an experimental FPS paradigm. Experimental studies assessing alterations in basic processes as a function of targeted brain stimulation in humans are highly valuable in determining how this treatment may alleviate disease. Based on the present findings, we can conclude that it is not at a basic level of defense that DBS affects symptoms of OCD.

## Conflict of Interest Statement

This DBS intervention was supported by an unrestricted investigator-initiated research grant by Medtronic, Inc. to Drs. Denys and Schuurman, which provided the devices used. Dr. Denys receives occasional fees from Medtronic for educational purposes. Dr. Schuurman is an independent consultant for Medtronic, Inc. on educational matters and received travel grants from the company. All other authors declare no conflict of interest.

## Footnotes

^1^www.digitimer.com

^2^www.biosemi.com

^3^www.neurobs.com

^4^www.brainproducts.com
